# Is it only humans that count from left to right?

**DOI:** 10.1098/rsbl.2009.0960

**Published:** 2010-01-13

**Authors:** Rosa Rugani, Debbie M. Kelly, Izabela Szelest, Lucia Regolin, Giorgio Vallortigara

**Affiliations:** 1 Centre for Mind/Brain Sciences, University of Trento, Corso Bettini 31, 38068 Rovereto, Italy; 2 Department of Psychology, University of Saskatchewan, Saskatoon, Canada; 3 Department of General Psychology, University of Padova, Italy

**Keywords:** avian brain, mental number line, domestic chick, Clark's nutcracker

## Abstract

We report that adult nutcrackers (*Nucifraga columbiana*) and newborn domestic chicks (*Gallus gallus*) show a leftward bias when required to locate an object in a series of identical ones on the basis of its ordinal position. Birds were trained to peck at either the fourth or sixth element in a series of 16 identical and aligned positions. These were placed in front of the bird, sagittally with respect to its starting position. When, at test, the series was rotated by 90° lying frontoparallel to the bird's starting position, both species showed a bias for identifying selectively the correct position from the left but not from the right end. The similarity with the well-known phenomenon of the left-to-right spatially oriented number line in humans is considered.

## 1. Introduction

As early as 1880, Galton showed that humans describe and think of numbers as being represented on a mental number line. Furthermore, some statements such as ‘Numerals are always pictured by me in a straight line from left to right’ (Galton 1880) suggest he had realized that the number line is usually oriented from left to right. Modern research provides evidence that number magnitude may be represented on a left-to-right oriented number line (Dehaene 1993).

It remains to be shown, however, whether the spatial orientation of the human mental number line is acquired culturally (i.e. it may be linked to writing and reading rules) or if it depends, at least in part, on biologically specific biases in the allocation of attention in extra-corporeal space. Here we provide some evidence for the latter hypothesis, by looking at the behaviour of non-human animals lacking any culture-specific bias in the exploration of visual space.

There are aspects of the mental number line in humans which rely on the ability to represent ordinal relations. Such ability requires mastering the rule that when one element is added to a given set, the new set becomes larger than the previous one and smaller than the next. Although this has been shown in some non-human animals (Davis & Bradford 1986; Dacke & Srinivasan 2008), a simpler and basic ability consists of identifying an object on the exclusive basis of its position in a series of identical objects. Such ability has been widely documented in non-human animals (e.g. Rugani Regolin & Vallortigara 2007) and may represent an ideal condition for investigations of the biological foundation of left–right biases in number line representation because of its intrinsic relationship with the spatial disposition of elements.

Here, we investigated two bird species, adult Clark's nutcrackers (*Nucifraga columbiana*), a widely employed animal model for the study of spatial cognition, and newborn domestic chicks (*Gallus gallus*), which offer the possibility of testing animals, with known experiential histories, at a very early age in a simple task aimed at documenting left–right biases in the identification of an object on the exclusive basis of its position in a series of identical objects.

## 2. Material and methods

### (a) Subjects

Subjects were 14 male domestic chicks (*Gallus gallus*) and six male and four female wild-caught Clark's nutcrackers (*N. columbiana*). Birds were trained to identify the fourth (*n* = 5 nutcrackers and *n* = 8 chicks) or the sixth (*n* = 5 nutcrackers and *n* = 6 chicks) element in a series of 16 fixed identical aligned elements, sagittally oriented with respect to the bird's starting position. Chicks a few hours old were caged in standard cages (28 × 40 × 32 cm) at controlled temperature (28–31°C) and humidity (68%). Food and water were available ad libitum. Testing began when they were 5 days old (because of yolk sac reserve chicks are unmotivated to peck for food reward before day 4 post-hatching). The nutcrackers were housed individually in large cages (48 × 48 × 73 cm) at a controlled temperature of 22°C. The colony was maintained on a 12 : 12 h light : dark cycle. The birds were maintained at 85 per cent of their free feeding weight. Water and grit were provided ad libitum.

### (b) Apparatus

The apparatus was randomly rotated within the larger experimental room, from one trial to other, to prevent the use of external cues.

Chicks were tested in a wooden square-shaped arena (80 × 80 × 40 cm). Two openings (7 × 11 cm) positioned at the midline of two opposing walls connected the arena with two starting boxes (7 × 11 × 11.5 cm) located outside the arena. Along the midline of the arena's floor was a series of 16 identical and aligned holes (2.5 cm in diameter), spaced 1.5 cm from one another, for an overall length of 62.5 cm (8 cm apart from the starting point and 29.5 cm from the side walls). All holes could be blocked by a sliding bar (115 × 4.5 × 3 cm) positioned underneath the apparatus and manoeuvred by the experimenter. The bar contained a small elongated groove (0.8 × 2 × 2 cm) filled with chick crumbs, which could be uncovered as it was positioned underneath the holes allowing the chick access to food.

Nutcrackers were tested in a square enclosure (140 × 140 × 61 cm). An entrance into the enclosure was situated at the centre of each wall (23 × 35 cm). Each entrance was covered with a plastic panel attached on the outside of the wall. The starting box was positioned flush against a predetermined entrance allowing the bird to enter into the enclosure. Sixteen aligned holes were centred along the midline of the enclosure. Adjacent holes were 5 cm apart from centre to centre. One sand-filled container was inserted in each of these holes.

## 3. Experimental procedures for chicks

### (a) Shaping

One day prior to testing, after being deprived of food for approximately 3 h, each chick underwent a shaping procedure in the experimental arena.

### (b) Training

For a total of 20 trials, in each trial, the chick was placed in the starting position and permitted to walk towards and peck at any position. Only one peck on each trial was allowed. A trial was considered correct when the chick pecked at the correct reinforced position. The trial was terminated after 180 s in the absence of a response. All chicks produced at least five correct responses across 20 valid trials and thus, progressed to the subsequent phases.

### (c) Re-training

On the morning following training, the chicks underwent re-training, to ascertain that they had learned the task. Learning criterion was three consecutive correct trials.

### (d) Training test

An hour after re-training each chick underwent a training test consisting of 20 consecutive trials. During each trial, the chick was allowed one peck. Only correct responses were reinforced. If no response occurred within 60 s, the trial was terminated. At the end of each trial, the chick was gently placed back at the starting box and after approximately 2 s it was given a new trial.

### (e) Testing

Two hours after completion of the training test, chicks underwent 20 consecutive test trials. A new and identical apparatus was used for testing. On the floor of the test apparatus was a series of 16 holes (all identical to those described for test 1) approximately 14 cm from and along one of the walls. Thus, the new test series was rotated by 90° when compared with the training series, and placed parallel in front of the chick's starting point (at about 61.5 cm).

## 4. Experimental procedures for nutcrackers

### (a) Shaping

Each daily session consisted of five trials. The correct position was indicated by a black container, whereas the remaining containers were white. The correct container was filled with sand upon which two pine seeds were placed. The birds were given 30 s to habituate to being in the starting box, after which the door was lifted to allow entrance into the arena. Once the bird attained the seeds on four of the five trials, training began.

### (b) Training

Each daily session consisted of five reinforced trials. Training was conducted over a series of 11 phases to ensure accurate responding. The learning criterion to progress to the next phase was four correct responses out of five trials.

In phases 1–3, the correct position was marked with a black container, making it distinct from the remaining white containers. During each successive training phase the pine seeds were progressively occluded by sand. Reliance on the visual cue of the black container was reduced during phases 4–8 by decreasing the number of trials during which the black container was present. By training phase 9, all containers were white. In phases 10 and 11, there were one and two non-rewarded trials, respectively. Non-rewarded trials were terminated once the bird made two choices or 5 min elapsed, which ever occurred first.

Birds received one daily training session until they completed 50 training sessions, after which, they received two training sessions per day (separated by approximately 2 h).

### (c) Testing

Testing began immediately upon successful completion of training (i.e. four of five correct trials on two consecutive training sessions). Twenty test sessions with six trials per session were given. Within the session, each bird received one test trial, one control trial and four baseline trials. During test trials, the two side walls were removed allowing the bird to enter the enclosure from the sides parallel to the row of the containers. In order to remain in the testing phase, the bird was required to obtain the correct position in the first attempt on three out of four baseline trials.

## 5. Analysis

The mean percentages ((number of pecks to a given position/20) × 100) of pecks made per test by the nutcrackers and by the chicks (either trained on the fourth or on the sixth position) to the correct positions (both from the left and from the right end of the test series) were computed. One-sample two-tailed *t*-tests were used to assess significant departures from chance level (6.250%, i.e. the probability to peck by chance at each of the 16 positions in a given trial, 100/16).

**Figure 1.**
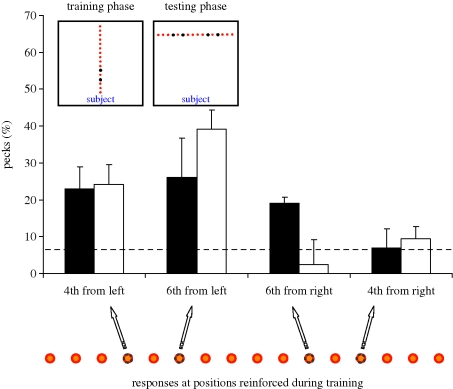
Left-side bias in birds in an ordinal task. Schematic illustration of the arena set up during training (top left) and during testing (top right), showing the orientation within the arena of the series of 16 positions with respect to the subject. The reinforced positions have been highlighted. The graph represents the mean percentages with s.e.m. ((number of pecks to a given position/20) × 100) of pecks emitted at test by the nutcrackers and by the chicks (either trained on the fourth or on the sixth position) to the correct positions (both from the left and from the right end of the test series). Filled bars, nutcrackers (*n* = 10); open bars, chicks (*n* = 14). Below the graph is shown the left–right-oriented test series, highlighting the reinforced positions during training.

Overall, at test, birds showed a left-hemispace bias—selecting the location from the left-hand side more often than from the right-hand side (figure 1).

The chicks selectively chose the correct position significantly above chance only when locating it from the left end: chicks trained on the fourth position (means ± s.e.m.: 24.125 ± 5.965, *t*(7) = 2.997; *p* = 0.020) or on the sixth position (means ± s.e.m.: 39.167 ± 10.833, *t*(5) = 3.025; *p* = 0.029). All other positions in the series were pecked either at or below chance level, even the correct positions from the right end: fourth position from right (means ± s.e.m.: 9.375 ± 5.039, *t*(7) = 0.620; *p* = 0.555), sixth position from right (means ± s.e.m.: 2.500 ± 1.708). Thus chicks showed a bias to choose the correct position from the left-hand side significantly more often than the correct position from the right-hand side.

The nutcrackers chose the correct position significantly above chance despite the rotation of the array by 90°. The fourth position from the left end was chosen significantly more than expected by chance (mean ± s.e.m.: 23.000 ± 5.385, *t*(4) = 3.111, *p* = 0.036), and so was the sixth position from the left end (mean ± s.e.m.: 26.000 ± 5.099, *t*(4) = 3.873, *p* = 0.018). Although the birds trained on the sixth position showed an increase in choices to the sixth position from the right, this was not significantly different from chance (mean ± s.e.m.: 19.000 ± 6.782, *t*(4) = 1.881, *p* = 0.133). All other positions were chosen either at or below chance level (including the fourth position from the right (mean ± s.e.m.: 7.000 ± 3.391, *t*(4) = 0.221, *p* = 0.836). Both groups of birds showed a bias for the container in the correct position located on the left-hand side. Thus, the birds were able to determine the correct container based on its ordinal position starting from the left-hand side.

## 6. Discussion

Both species performed successfully at test, when some of the non-numerical cues available during training, such as distance from the starting point and walking time, could not be employed to solve the task (although they could still rely on the distance of the correct position from the beginning of the series). Interestingly, when locating an object in a series of identical objects on the basis of its ordinal position both species showed a leftward bias. Our results indicate for the first time that a disposition to map the numerical number line from left to right exists in non-human, non-linguistic species, possibly as a result of right hemispheric dominance in visuospatial tasks, resulting in the left visual hemifield controlling the birds' behaviour (Diekamp *et al*. 2005; Regolin 2006; and for similarity with humans see Vallortigara & Rogers 2005). The results with chicks support the fact that such a disposition is apparent very early in development.

As stated in §1, however, the parallel with the human condition should be considered with caution. Effects associated with the left-to-right orientation in the mental number line in humans (such as the *Spatial Numerical Association of Response Codes*—SNARC effect, Dehaene 1993) are linked to magnitude estimation, which is clearly absent in our task. This aspect may be specific for humans only. Further comparative research, with more complex ordinal tasks involving magnitude estimation is needed.
